# Anti-inflammatory effect and mechanism of action of *Lindera erythrocarpa* essential oil in lipopolysaccharide-stimulated RAW264.7 cells

**DOI:** 10.17179/excli2017-596

**Published:** 2017-08-29

**Authors:** Yeong-Jong Ko, Ginnae Ahn, Young-Min Ham, Sang-Mock Song, Eun-Yi Ko, Su-Hyeon Cho, Weon-Jong Yoon, Kil-Nam Kim

**Affiliations:** 1 Jeju Biodiversity Research Institute (JBRI), Jeju Technopark (JTP), Jeju 699-943, Republic of Korea; 2 Department of Marine Bio-Food Sciences, Chonnam National University, Yeosu 550-74, Republic Korea; 3 Chuncheon Center, Korea Basic Science Institute (KBSI), Chuncheon 200-701, Republic of Korea; 4 Department of Marine Biotechnology, University of Science and Technology, Daejeon 305-350, Republic of Korea

**Keywords:** Lindera erythrocarpa, anti-inflammatory, essential oil, NF-kappaB, MAPK

## Abstract

The aim of this study was to investigate the chemical constituents of *Lindera erythrocarpa* essential oil (LEO) by gas chromatography-mass spectrometry and evaluate their inhibitory effect on the expression of pro-inflammatory mediators in lipopolysaccharide (LPS)-stimulated RAW264.7 cells. Fifteen compounds, accounting for 63.7 % of the composition of LEO, were identified. The main compounds were nerolidol (18.73 %), caryophyllene (14.41 %), α-humulene (7.73 %), germacrene-D (4.82 %), and α-pinene (4.47 %). LEO significantly inhibited the expression of inducible nitric oxide (NO) synthase and cyclooxygenase-2, and subsequent production of NO and prostaglandin E_2_. In addition, it reduced the release of pro-inflammatory cytokines in LPS-activated RAW264.7 cells. The molecular mechanism underlying the effect of LEO was associated with inhibition of the phosphorylation of mitogen-activated protein kinase (MAPK). Furthermore, LEO inhibited LPS-induced phosphorylation and degradation of inhibitor of kappa B-α, which is required for the activation of the p50 and p65 nuclear factor (NF)-κB subunits in RAW264.7 cells. Taken together, these data suggest that LEO exerted its anti-inflammatory effect by downregulating LPS-induced production of pro-inflammatory mediators through the inhibition of NF-κB and MAPK signaling in RAW264.7 cells.

## Introduction

Prolonged inflammation is involved in the pathogenesis of a variety of diseases, including pulmonary fibrosis, atherosclerosis, chronic hepatitis, rheumatoid arthritis, and inflammatory brain diseases (Chung et al., 2007[7]). Inflammation is caused by pro-inflammatory mediators, including both pro-inflammatory cytokines such as tumor necrosis factor (TNF)-α, interleukin (IL)-1β, and IL-6, and pro-inflammatory factors such as nitric oxide (NO), prostaglandin E_2_ (PGE_2_), inducible nitric oxide synthase (iNOS), and cyclooxygenase (COX)-2 (Ljung et al., 2006[24]; Walsh et al., 2005[39]). Therefore, inhibition of these inflammatory mediators is an important target pathway in the treatment of diseases with anti-inflammatory components. 

Nuclear transcription factor kappa-B (NF-κB) regulates a variety of genes associated with immune and acute-phase inflammatory responses. The activation of NF-κB due to pro-inflammatory stimulation is indicated by rapid phosphorylation and degradation of inhibitors of kappa B (IκBs) (Rajapakse et al., 2008[30]). Freed NF-κB dimers from this process translocate to the nucleus and bind to the promoter regions of target genes (Lee et al., 2003[21]). They then induce the transcription of pro-inflammatory mediators such as iNOS, COX-2, IL-6, IL-1β, and TNF-α (Makarov, 2000[25]; Yoshimura, 2006[44]). Recent studies have reported that the anti-inflammatory effect of phytochemicals occurs through blocking of the NF-κB signaling pathway (Ham et al., 2015[11]; Hsieh et al., 2011[14]). Mitogen-activated protein kinases (MAPKs) are one of the major kinases involved in cellular processes such as apoptosis, stress responses, differentiation, and immune defense (Liu et al., 2007[23]). MAPKs comprise three major subgroups, namely, p38 MAPKs, c-Jun N-terminal kinases (JNKs), and extracellular signal-regulated kinases (ERKs). Their activation plays an important role in the expression of iNOS and COX-2 and in the production of cytokines (Rajapakse et al., 2008[30]; Rao, 2001[31]). Therefore, MAPK and NF-κB may be effective as anti-inflammatory agents.

*Lindera erythrocarpa* is widely distributed in the Republic of Korea, Japan, and China (Sun and Chung, 1988[35]). *Lindera* species, including *L. strychnifolia*,* L. lucida*,* L. chunii*, and *L. aggregate* are important medicinal plants. The fruit and leaves of *L. erythrocarpa* are used in folk medicine for treating digestive disorders, thirst, pain, and neuralgia. They are also used as antidotes and diuretics (Hong et al., 2009[13]; Oh et al., 2005[28]; Sun and Chung, 1988[35]). Recently, *L. erythrocarpa* was reported to suppress adipogenesis and melanin synthesis, attenuate obesity, as well as exhibit antioxidant, anti-inflammatory, and antifungal activities (Hsieh and Wang, 2013[15]; Hwang et al., 2007[16]; Kumar et al., 2010[19]; Wang et al., 2008[40]). Essential oils extracted from medicinal and aromatic plants are known to have biological effects, most notably anti-inflammatory, antioxidant, antifungal, and antibacterial activities (Chaieb et al., 2007[4]; Pinheiro et al., 2011[29]). These properties are a driving interest in the use of essential oils in the cosmetic, pharmaceutical, and food industries (Chaieb et al., 2007[4]; Lang and Buchbauer, 2012[20]; Tumen et al., 2010[37]). However, the mechanism by which *L. erythrocarpa *essential oil (LEO) exerts its anti-inflammatory effect has not been elucidated. Therefore, in this study, we examined the anti-inflammatory effects of LEO and its constituents on lipopolysaccharide (LPS)-stimulated RAW264.7 cells.

## Materials and Methods

### Reagents

LPS, phosphate buffered saline (PBS), dimethyl sulfoxide (DMSO), 3-(4,5-dimethylthiazol-2-yl)-2,5-diphenyltetrazolium bromide (MTT) and radio-immunoprecipitation assay RIPA lysis buffer were bought from Sigma-Aldrich (St. Louis, MO, USA). Fetal bovine serum (FBS) and Dulbecco's modified Eagle's medium (DMEM) were purchased from Invitrogen-Gibco (Grand Island, NY, USA). Enzyme-linked immunosorbent assay (ELISA) kits for the analyses of TNF-α, IL-6, and PGE_2_ were obtained from BD Biosciences (San Diego, CA, USA) and R&D Systems, Inc. (St. Louis, MO, USA). Antiphosphorylated IκB-α (anti-p-IκB-α), anti-NF-κB, anti-JNK, anti-p38, anti-ERK1/2, anti-phosphorylated JNK (anti-p-JNK), anti-phosphorylated p38 (anti-p-p38) and anti-phosphorylated ERK1/2 (anti-p-ERK1/2) mouse or rabbit antibodies were bought from Cell Signaling Technology (Beverly, MA, USA). All other reagents were obtained from Sigma-Aldrich.

### Extraction of essential oil from the leaves of L. erythrocarpa

The leaves of *L. erythrocarpa* were collected from Namwon (a region in Jeju Island, Korea) in May 2014 and LEO was extracted by hydrodistillation. Briefly, approximately 300 g of fresh *L*.* erythrocarpa* leaves was immersed in 3 l of distilled water in a 5-l flask. Subsequently, the obtained essential oil was dried over anhydrous sodium sulfate, filtered, and stored in a sealed vial at 4 °C until tested. The LEO yield was approximately 0.067 % (v/w).

### Gas chromatography (GC)-mass spectrometry (MS) analysis

Analysis of the main components of the most active essential oil extracted from *L*.* erythrocarpa* leaves was carried out using a GC (Agilent 6890, Agilent Technologies Inc., Santa Clara, CA, USA) connected to an MS (Agilent 5975). The GC was equipped with a DB1-HT column (30 m × 0.32 mm; 0.1 µm film thickness). The oven temperature was programmed to increase from 40 to 100°C at a rate of 2 °C/min, and then from 100 to 230 °C at a rate of 5 °C/min, after which it was held at 230 °C for 5 min. The detector and injector temperatures were 280 °C and 240 °C, respectively. The flow rate of the carrier gas (He) was 1.5 ml/min and the split ratio was 1:10. For the sample injection (split less), 10 μl of LEO was diluted in 500 μl of CH_2_Cl_2_ and 1 μl of this solution was injected for analysis. Identification of the compounds was done by comparison of their mass spectra with reference spectra in the Wiley libraries. The GC-MS retention indices were also calculated using a homologous series of n-alkanes (C6-C31).

### Cell culture

The murine macrophage cell line RAW264.7 (Korean Cell Line Bank, Seoul, Korea) was cultured in DMEM (100 U/ml penicillin, 100 μg/ml streptomycin and 10 % FBS) in an atmosphere of 5 % CO_2_ at 37 °C and were subcultured every 3 days.

### Cell viability assay

The cytotoxicity of LEO against RAW264.7 cells was examined via an MTT assay. RAW264.7 cells (1.8 × 10^5^ cells/ml) were plated in 96-well plates for 24 h and then treated with aliquots of LEO at 37 C for 24 h. MTT solution was added to each well for 4 h. The plates were centrifuged at 2000 rpm for 10 min, and the supernatants were removed by aspiration. The formazan crystals in each well were dissolved in DMSO and absorbance was measured at 540 nm.

### Determination of NO production

RAW264.7 cells (1.8 × 10^5^ cells/ml) were plated in 24 well-plates for 24 h and then cultured with LPS (1 μg/ml) in the absence or presence of LEO (0.01 %, 0.02 %, and 0.04 %) for 24 h. The amount of nitrite that accumulated in the culture medium was measured and used as an indicator of NO production. Briefly, 100 μl of cell culture medium was mixed with an equal volume of 1 X Griess reagent, and incubated at room temperature for 10 min. Absorbance was then measured at 540 nm. A fresh culture medium was used as the blank in every experiment. 

### ELISA assay 

Sandwich ELISA was used to determine the inhibitory effects of 0.01 %, 0.02 %, and 0.04 % of LEO on the production of PGE_2_, TNF-α, and IL-6 in LPS-stimulated RAW264.7 cells. The analyses were done using the relevant ELISA kits according to the manufacturer's instructions. The cells were stimulated for 24 h before the supernatant was harvested and assayed.

### Western blot analysis 

RAW264.7 cells were treated with LPS in the absence or presence of the aforementioned concentration of LEO. After incubation, the cells were washed twice with cold PBS and lysed in RIPA lysis buffer. The cell lysates were then isolated by centrifugation at 15,000 rpm for 15 min at 4 ^°C. Protein concentration in the supernatants was measured by the Bradford assay (Bio-Rad, Hercules, CA, USA) and all proteins were adjusted to an equal protein content. Aliquots of the lysates (20~30 μg protein/lane) were separated on a NuPAGE 4-12 % bis-Tris gel (Invitrogen, Carlsbad, CA, USA) and the separated proteins were transferred onto polyvinylidene difluoride (PVDF) membranes using an iBlot gel transfer device (Invitrogen). The membranes were immersed in 5 % non-fat skim milk, which was used as a blocking solution and incubated with primary antibodies (1:1,000) at 4 °C for 24 h. After incubating, the membranes were washed several times with Tween 20-Tris-buffered saline and incubated with secondary horseradish peroxidase-linked anti-rabbit or anti-mouse IgG (1:5,000, Cell Signaling Technology) for 90 min. After washing, immunoactive proteins were detected using the WEST-ZOL (plus) Western Blot Detection System (iNtRON Biotechnology, Gyeonggi, Korea).

### Statistical analysis 

All data have been reported as mean ± standard deviation. Statistical analyses of the data were using Student's *t*-test. P values less than 0.05 were considered to be statistically significant.

## Results

### Chemical composition and cytotoxicity of LEO

**Figure 1:**
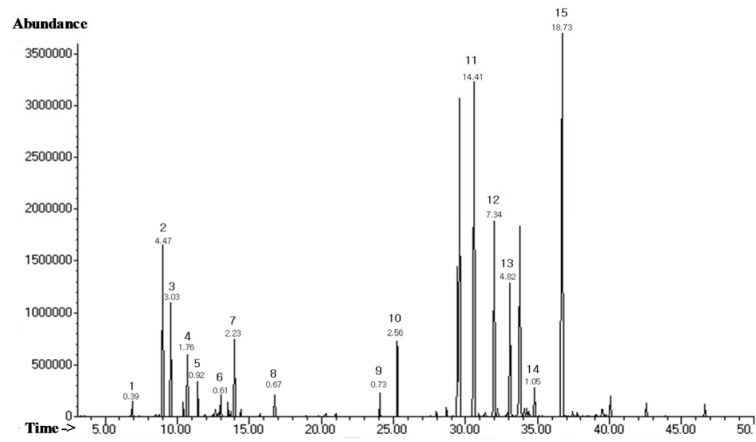
GC-MS total ion chromatogram of the essential oil of LEO. The compound labels in the chromatogram correspond to the Arabic numerals given in Table 1.

**Figure 2:**
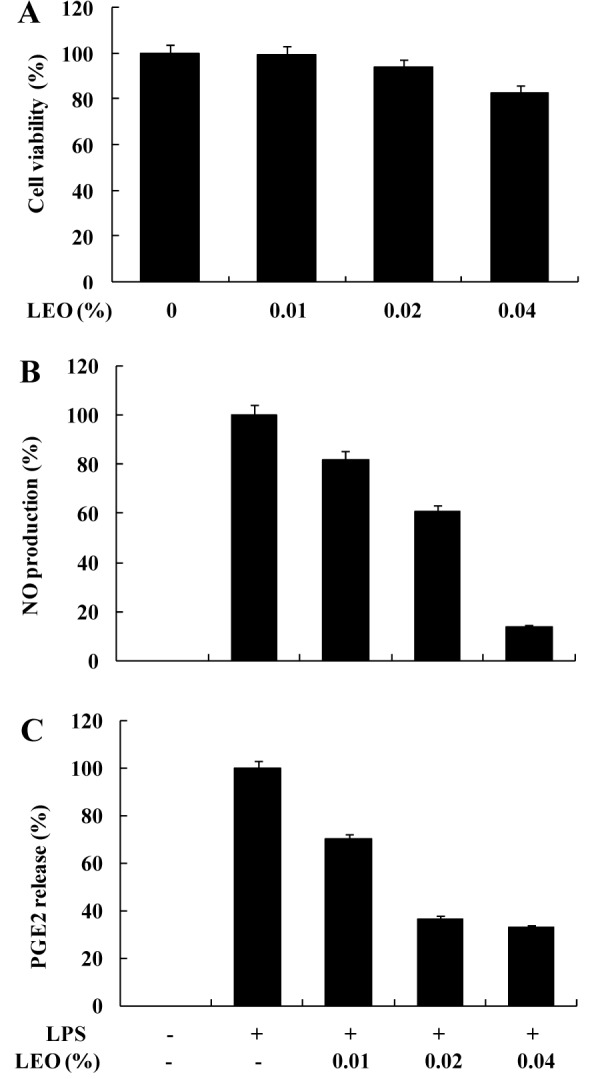
Effect of LEO on NO and PGE_2_ production in LPS-induced RAW264.7 cells. Cells (1.8 × 10^5^ cells/ml) were stimulated by LPS (1 μg/μl) for 24h in the presence of LEO (0.01, 0.02, and 0.04 %). (A) Cytotoxicity of LEO was assessed by MTT. Supernatants were collected, and the (B) NO and (C) PGE_2_ concentration in the supernatants was determined using Griess reaction and the enzyme immunoassay, respectively. Values are expressed as means ± S.D. of triplicate experiments.

The chemical composition and retention indices of the constituents of LEO are presented in Table 1(Tab. 1) and Figure 1(Fig. 1). A total of 15 volatile constituents were identified, representing 63.73 % of the composition of LEO. The main constituents of LEO were nerolidol (18.73 %), caryophyllene (14.41 %), α-humulene (7.73 %), germacrene-D (4.82 %), and α-pinene (4.47 %). We initially conducted a cytotoxicity test via an MTT assay (Figure 2A(Fig. 2)) and found that LEO (0.01, 0.02, and 0.04 %) was not cytotoxic to the RAW264.7 cells; therefore, we used the oil at concentrations of 0.01-0.04 % in subsequent experiments.

### Effects of LEO on the production of NO and PGE_2_ in LPS-stimulated RAW264.7 cells

We investigated the potential anti-inflammatory effect of LEO by evaluating the production of NO and PGE_2_ in LPS-stimulated RAW264.7 cells. As shown in Figure 2B(Fig. 2), NO production was substantially higher in LPS-treated cells than in the untreated cells. However, LEO suppressed NO production in the LPS-treated cells in a dose-dependent manner. Addition of 0.04 % LEO to the cells caused a reduction in LPS-induced NO production by 86.1 %. Moreover, results of the PGE_2_ assays revealed a significant dose-dependent inhibition of PGE_2 _production by LEO in the LPS-activated cells. PGE_2_ production was inhibited by 54.1, 65.8, and 71.8 % by LEO at concentrations of 0.01, 0.02, and 0.04 %, respectively (Figure 2C(Fig. 2)).

### Effects of LEO on the expression of iNOS and COX-2 in LPS-stimulated RAW264.7 cells

**Figure 3:**
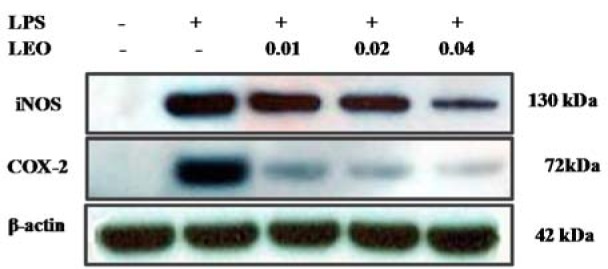
Effects of LEO on production of iNOS and COX-2 protein in LPS-induced RAW264.7 cells. RAW264.7 cells (1.8 × 10^5^ cells/ml) were pre-incubated for 18 h, and the cells were stimulated with LPS (1 μg/μl) in the presence of LEO (0.01, 0.02, and 0.04 %) for 24 h. iNOS and COX-2 protein levels were determined via Western blot method.

Western blot analyses were used to determine whether the inhibitory activity of LEO on the production of NO and PGE_2_ was related to the expression levels of iNOS and COX-2. The data shown in Figure 3(Fig. 3) demonstrate that the expression levels of iNOS and COX-2 in the LPS-stimulated cells increased when compared with the untreated control. However, LEO (0.01, 0.02, and 0.04 %) inhibited LPS-induced increases in iNOS and COX-2 levels in a dose-dependent manner. These results are consistent with the inhibitory effects of LEO on the production of NO and PGE_2_.

### Effects of LEO on the production of IL-6 and TNF-α in LPS-stimulated RAW264.7 cells

**Figure 4:**
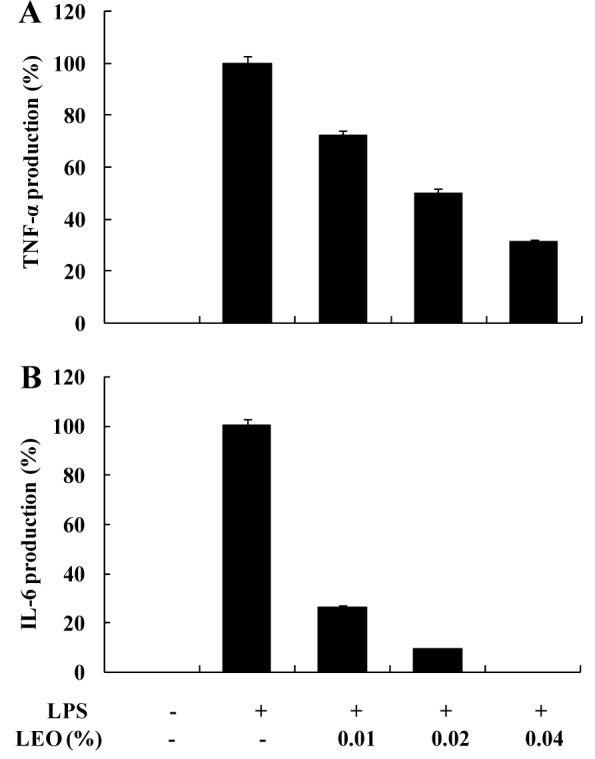
Effect of LEO on IL-6 and TNF-α production in LPS-induced RAW264.7 cells. Cells (1.8 × 10^5^ cells/ml) were stimulated by LPS (1 μg/μl) for 24 h in the presence of LEO (0.01, 0.02, and 0.04 %). Supernatants were collected, and the IL-6 and TNF-α concentration in the supernatants was determined by ELISA. Values are expressed as means ± S.D. of triplicate experiments.

We further investigated the effects of LEO on the secretion of IL-6 and TNF-α in LPS-activated RAW264.7 cells. As shown in Figure 4(Fig. 4), the production of IL-6 and TNF-α was considerably increased by LPS but significantly inhibited by LEO in a dose-dependent manner. 

### Effect of LEO on the NF-κB signaling pathway in LPS-activated RAW264.7 cells

**Figure 5:**
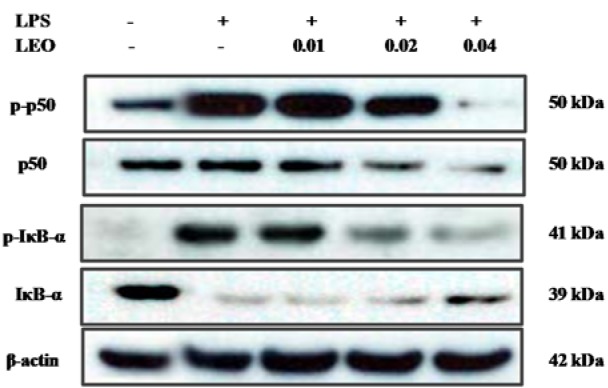
Effect of LEO on phosphorylation of NF-κB in LPS-induced RAW264.7 cells. RAW264.7 cells (1.8 × 10^6^ cells/ml) were pre-incubated for 18 h, and the cells were stimulated with LPS (1 μg/μl) in the presence of LEO (0.01, 0.02, and 0.04 %) for 30 min. The levels of p-p50, p 50, p-IκB-α and IκB-α were determined using Western blot method.

Since the phosphorylation of IκB and its subsequent degradation are essential steps in NF-κB activation by LPS, we examined the effect of LEO on these processes in a Western blot analysis. Our results showed that LEO significantly decreased the phosphorylation and degradation of IκB-α in the LPS-activated cells (Figure 5(Fig. 5)). NF-κB plays a critical role in the inflammatory response; therefore, we further investigated whether LEO blocks NF-κB activation in LPS-stimulated cells. Our results showed that LEO dose-dependently inhibited LPS-induced NF-κB activation. These findings suggest that LEO attenuates NF-κB activation by inhibiting IκB-α degradation, which further inhibits the expression of inflammatory mediators. 

### Effects of LEO on the phosphorylation of MAPKs in LPS-activated RAW264.7 cells

**Figure 6:**
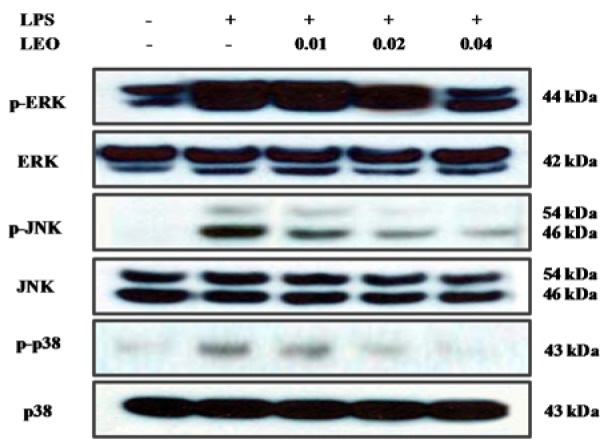
Effect of LEO on phosphorylation of MAPK in LPS-induced RAW264.7 cells. RAW264.7 cells (1.8 × 10^6^ cells/ml) were pre-incubated for 18 h, and the cells were stimulated with LPS (1 μg/μl) in the presence of LEO (0.01, 0.02, and 0.04 %) for 30 min. The levels of p-ERK, ERK, p-JNK, JNK, p-p38 and p38 were determined using Western blot method.

We investigated by Western blotting whether inhibition of the expression of pro-inflammatory mediators by LEO is mediated by the MAPK pathway. The results indicated that LEO inhibited LPS-induced phosphorylation of JNK, p38, and ERK in a concentration-dependent manner (Figure 6(Fig. 6)). These findings suggest that the anti-inflammatory activity of LEO is mediated by inhibition of LPS-induced phosphorylation of MAPKs.

## Discussion

The results of the study demonstrate that LEO exhibits significant inhibitory effects on the expression of pro-inflammatory mediators such as NO, PGE_2_, iNOS, COX-2, IL-6, and TNF-α. These effects were accompanied by inhibition of the phosphorylation of MAPKs and NF-κB. 

Nerolidol, the major component of the essential oil of *Peperomia serpens*, has been shown to inhibit carrageenan-induced paw and ear edema in mice (Pinheiro et al., 2011[29]). Fonseca et al. (2016[10]) demonstrated the anti-inflammatory effect of nerolidol via its inhibitions of carrageenan-induced paw edema formation and LPS-stimulated IL-6 and IL-1β production in peritoneal macrophages. Furthermore, the anti-inflammatory activities of α-humulene and (-)-trans-caryophyllene have been demonstrated via their inhibition of the production of different inflammatory mediators in carrageenan-treated rats (Fernandes et al., 2007[9]). In addition, other minor compounds present in LEO, such as α-pinene, β-pinene, β-myrcene, dl-limonene, geraniol, and bornyl acetate, have been shown to exhibit anti-inflammatory activities (Chen and Viljoen, 2010[6]; d'Alessio et al., 2013[8]; Rufino et al., 2014[33], 2015[34]; Wu et al., 2004[41]). Therefore, the anti-inflammatory activity of LEO could be attributed to its major constituents or a synergetic action among the constituents present in the oil.

Macrophages are activated by LPS produce pro-inflammatory factors, such as NO, PGE_2_, iNOS, and COX-2 (Korhonen et al., 2005[18]). For this reason, we selected LPS as an inflammatory stimulant for the study. The production of NO by iNOS due to stimulation with LPS plays an important role in inflammation (McCann et al., 2005[26]). Similar to NO, PGE_2_ is an inflammatory mediator generated at inflammatory sites by COX-2, which is involved in the pathogenesis of many chronic inflammatory diseases, such as rheumatoid arthritis, cancer, and cardiovascular disease (Lipsky, 1999[22]; Rocca and FitzGerald, 2002[32]). Thus, reducing the levels of NO, PGE_2_, iNOS, and COX-2 may be an attractive target for confirming anti-inflammatory effects. In addition, the pro-inflammatory cytokines IL-1β, IL-6, and TNF-α are known to contribute to inflammatory diseases, such as pulmonary fibrosis, atherosclerosis, chronic hepatitis, rheumatoid arthritis, and inflammatory brain diseases (Chung et al., 2007[7]). Hence, we measured the effects of LEO on the expression of iNOS and COX-2 and the production of NO, PGE_2_, IL-6, and TNF-α in LPS-stimulated RAW264.7 cells. Pretreatment of the cells with LEO inhibited the production of NO and PGE_2_ by reducing LPS-induced expression of iNOS and COX-2. Moreover, LEO reduced LPS-induced TNF-α and IL-6 production. Other studies have similarly reported that the anti-inflammatory effect of essential oils is associated with the suppression of mediators such as PGE_2_, NO, IL-6, TNF-α, COX-2, and iNOS (Yoon et al., 2010[42][43]). 

MAPKs are the major components of the signaling pathway that leads to the expression of pro-inflammatory mediators. Moreover, it is known that MAPKs play an important role in inflammatory signaling in LPS-stimulated macrophages (Hommes et al., 2003[12]; Nakano et al., 1998[27]). Furthermore, p38 is involved in the transcriptional regulation of pro-inflammatory mediators, including iNOS, COX-2, and TNF-α, in LPS-induced macrophage responses (Bhat et al., 1998[3]). In addition, the phosphorylation of ERK is thought to be implicated in the increased production of pro-inflammatory cytokines and iNOS in LPS-induced macrophage responses (Ajizian et al., 1999[1]; Bhat et al., 1998[3]). Other studies have reported that JNK is primarily implicated in the expression of iNOS and COX-2 in LPS-stimulated macrophages (Chan and Riches, 1998[5]; Uto et al., 2005[38]). Therefore, we assessed the MAPK signaling pathway to elucidate the mechanism underlying the anti-inflammatory effect of LEO in LPS-activated macrophages. The results showed that pretreatment with LEO resulted in a significant suppression of JNK, ERK, and p38 phosphorylation in the LPS-activated macrophages.

NF-κB is a mammalian transcription factor that regulates many genes, including those involved the expression of iNOS, COX-2, TNF-α, IL-1β, and IL-6, which are important in immune and inflammatory responses in LPS-stimulated macrophages (Barnes and Karin, 1997[2]). In unstimulated cells, NF-κB is present as a quiescent form bound to the inhibitory protein IκB in the cytosol (Karin and Ben-Neriah, 2000[17]). Upon stimulation with LPS, IκB is degraded, which permits the nuclear translocation of the NF-κB complex, where it binds kb sites and activates the gene transcription of iNOS, COX-2, and other pro-inflammatory cytokines (Surh et al., 2001[36]). In our studies, LEO attenuated IκB-α degradation and suppressed NF-κB activation in LPS-stimulated RAW264.7 cells. This suggests that it inhibits the activation of inflammatory mediators by blocking the NF-κB signaling pathway during the activation of macrophages.

In conclusion, the anti-inflammatory activity of LEO is evidenced by its inhibition of the production of NO, PGE_2_, and other cytokines (e.g., TNF-α and IL-6) via regulation of the NF-κB and MAPK pathways. These results suggest that the development of substances that modulate the expression of pro-inflammatory mediators can provide a molecular basis for developing functional foods for the treatment of various inflammatory diseases. 

## Notes

Yeong-Jong Ko and Ginnae Ahn contributed equally to this work as first authors.

Weon-Jong Yoon and Kil-Nam Kim (Chuncheon Center, Korea Basic Science Institute (KBSI), Chuncheon 200-701, Republic of Korea; Tel.: +82-33-815-4607, E-mail: knkim@kbsi.re.kr) contributed equally as corresponding authors.

## Conflict of interest

The authors have no competing financial interests to declare.

## Acknowledgements

This work was supported by the Korea Basic Science Institute (C37965) and (C37260).

## Figures and Tables

**Table 1:**
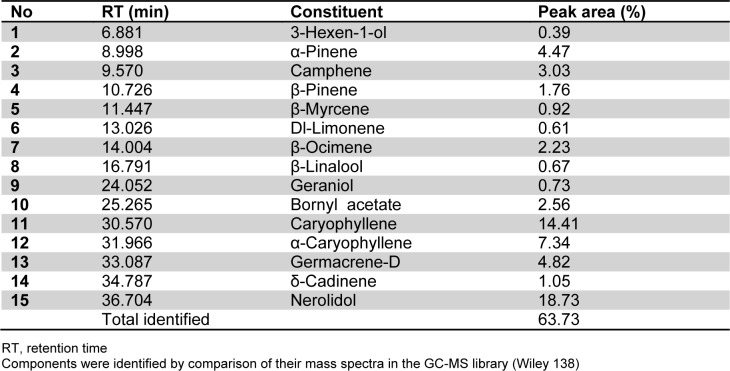
Chemical composition of the essential oil from *Lindera erythrocarpa*
